# The role of supply responses in public insurance expansion: evidence from the New Cooperative Medical Scheme in China

**DOI:** 10.1186/s12913-025-13090-0

**Published:** 2025-07-16

**Authors:** Lin Lin, Xianhua Zai

**Affiliations:** 1 School of Public Management, East China Normal University, Shanghai, China; 2 Department of Labor Demography, Max Planck Institute for Demographic Research, 1 Konrad-Zuse-Str, 18057 Rostock, Germany; 3 Max Planck – University of Helsinki Center for Social Inequalities in Population Health, Helsinki, Finland

**Keywords:** Health insurance in China, NCMS, Supply responses, Medical resources, H51, I12, I13, I18

## Abstract

In our study, we explore the relationship between the New Cooperative Medical Scheme (NCMS) — a significant health insurance expansion in rural China — and healthcare supply, utilizing a nationwide dataset spanning from 2004 to 2011. Our findings reveal notable positive supply-side responses to the NCMS insurance expansion, characterized primarily by a substantial increase in the number of inpatient beds at township health centers serving rural residents. Moreover, we estimate large increases in the number of county hospitals; however, these effects lack statistical significance. These findings indicate that the surge in demand prompted by NCMS enrollment gain is associated with greater healthcare investment by existing rural providers rather than encouraging new providers to enter the market. Furthermore, when adjusting for these supply-side responses, we find that responsive healthcare supply plays a crucial role in mediating the effectiveness of the NCMS: the growth in inpatient care utilization following the NCMS is significantly mitigated once the availability of inpatient beds is controlled. These insights underscore the universal importance of responsive healthcare supply in the effectiveness of public insurance expansions.

**Supplementary Information:**

The online version contains supplementary material available at 10.1186/s12913-025-13090-0.

## Introduction

Aligned with the World Health Organization’s advocacy, Low- and Middle-Income Countries (LMICs) have been progressively expanding health insurance coverage to their uninsured low-income populations since the 1990s,^1^ aiming to achieve the goal of universal health insurance coverage. Contrary to the experience in high-income nations that public health insurance programs have been consistently effective in improving healthcare utilization and health outcomes,^2^ public insurance initiatives in several LMICs have been shown to have limited or no significant impact.^3^ Therefore, it is essential to investigate the factors influencing the success of public insurance policies in LMICs to enhance their efficacy.

Examining successful experiences in high-income countries, supply-side responses have played a significant role in the success of public insurance reforms in increasing healthcare utilization and improving health outcomes. Increased medical investments by hospitals can triple the inpatient effect of public insurance expansion in the United States [5]. Similarly, Japan’s introduction of universal health insurance in 1961 significantly increased the number of hospital beds to meet the rising healthcare demand [24]. In contrast, limited healthcare supply in LMICs may constrain the effectiveness of health insurance expansions. In LMICs, the post-reform increase in healthcare demand may be restricted by the less generous coverage provided under public insurance schemes. The demand shock is further weakened by the disadvantaged economic conditions of the targeted low-income population.^4^ Consequently, the healthcare supply side in LMICs is regarded as less likely to respond as effectively to increased healthcare demand following insurance expansion as it does in high-income countries.

However, limited research has been conducted in LMIC settings to examine whether, and to what extent, supply-side responses are associated with increased healthcare utilization. A related study documented an increase in admissions and inpatient days following the expansion of universal health insurance in Vietnam [25]. However, the magnitude of this increase was smaller than that observed in high-income countries. The authors suggest that the limited responsiveness of healthcare resources in lower-level medical institutions may constrain the effectiveness of insurance expansion. Nevertheless, more rigorous research with stronger identification strategies is needed in more LMIC settings to address key questions such as: Are there sufficient supply-side responses following public insurance expansions in LMICs? To what extent can a responsive healthcare supply—if present—enhance the effectiveness of insurance expansion? Answering these questions is crucial for governments seeking to expand or improve their insurance programs, especially in resource-constrained settings, yet it remains understudied in LMICs due to insufficient supply-side data [26].

Our paper attempts to fill these gaps by utilizing China’s New Cooperative Medical Scheme (NCMS) reforms as a natural experiment to explore the supply-side effects of public insurance reforms in LMICs. Initiated in 2003, the NCMS was designed to extend health insurance coverage to China’s rural population, which largely comprised individuals without formal employment and, consequently, without access to urban employee health insurance before the reform. Prior to the NCMS, around 82% of rural inhabitants lacked health insurance. The scheme was rolled out on a county-by-county basis and expanded rapidly, ultimately providing coverage to 640 million previously uninsured rural residents by 2008-representing 95% of China’s rural demographic. Our previous work [27], which examined the impact of the NCMS expansion on healthcare utilization, has showed the effectiveness of the NCMs in improving healthcare use. In this study, we extend the analysis by focusing on the supply-side responses to the increased demand generated by NCMS enrollment.

In rural China, the majority of healthcare is delivered by public providers. Healthcare professionals are typically salaried employees of public health centers or hospitals, making medical institutions responsible for supply decisions, including the hiring of doctors and ancillary staff, as well as investments in hospital facilities, medical equipment, and beds. Given the supply side’s discretion in decision-making, we first examine whether and to what extent healthcare supply responds to the rural health insurance expansion of the NCMS. The substantial addition of hundreds of millions of new beneficiaries is expected to create strong incentives for healthcare providers to expand their supply. To assess whether the supply-side responses, if any, are adequate, we compare the magnitude of our estimates to the benchmark supply-side effects as estimated in studies from high-income countries. Should our supply-side effects be outpaced, this could suggest that a restrained supply-side response may be contributing to the weaker efficacy for China’s NCMS reform. Furthermore, we quantify the role of supply-side responses following the NCMS to the scheme’s efficacy using mediation analysis.

Our study focuses on supply-side measures that have not been explored extensively in previous studies, such as the number of inpatient beds and medical institutions, using the province-level panel data in 2004–2011. The identification relies on the plausibly exogenous temporal and geographical variations in NCMS enrollment rate in a two-way fixed effect model conditional on socio-economic characteristics of each province. We find that the supply-side responses to NCMS enrollment differ across types of healthcare resources. While NCMS enrollment is associated with an increase in the number of medical providers in areas with larger demand shocks, the estimates lack statistical significance. Conversely, the number of inpatients beds increases significantly in response to the increase in insurance enrollment, particularly at township health centers. The distinct impact observed in the expansion of inpatient beds, as opposed to the more modest growth in healthcare facilities, could be attributed to the lower cost associated with augmenting existing infrastructure compared to the substantial fixed expenses required for establishing new healthcare centers or hospitals. To address concerns that the supply-side responses might be resulted from increased government subsides to rural healthcare organizations in 2009–2011 [28], we present the dynamic effects of NCMS enrollment on healthcare resources and show the increase in hospital beds is mainly driven in years before 2009.

To contextualize our findings within the broader LMIC landscape, the supply-side responses we estimate are greater than those documented in Vietnam, both in magnitude and scope. As shown by [25], the supply-side response in Vietnam was limited to higher-level hospitals. In contrast, our findings reveal that supply-side responses occurred in lower-level medical institutions serving the targeted beneficiaries. In previous studies [27], documented a significant increase in healthcare utilization following the NCMS expansion in China, whereas [25] reported a smaller increase in inpatient care use in Vietnam. Taken together, this evidence suggests that a more responsive healthcare supply side is crucial in enhancing the effectiveness of health insurance expansion. Moreover, the increase in inpatient beds following the NCMS implementation, as identified in this study, is comparable to supply-side responses observed in high-income countries. This provides additional evidence that the healthcare supply side in rural China has adequately adapted to the demand surge generated by the insurance expansion.

Furthermore, we investigate the extent to which the supply-side responses can mediate the relationship between the NCMS expansion and healthcare utilization. After accounting for hospital beds, we find that the magnitude of NCMS enrollment’s effect on inpatient care use is reduced by about 50%. This suggests that approximately half of the success of the NCMS in increasing healthcare utilization is associated with concurrent supply-side responses aimed at meeting increased healthcare demand. These findings suggest that a less responsive supply side may not be contributing to the limited efficacy of insurance expansion in LMICs. Rather, the efficacy of health insurance expansion in LMICs would require demand-side supporting policies such as improved insurance benefits.

### Related literature

The first relevant branch of literature comprises studies on the effects of the NCMS. On the one hand, some of these studies find that the NCMS is not effective in terms of increasing outpatient care use [29–31].^5^ On the other hand, more studies find evidence in support of the effectiveness of the NCMS in terms of increasing healthcare use, providing financial protection, and improving health outcomes.^6^ Our study contributes to this branch of literature by exploring the supply-side mechanism behind its effectiveness, and suggesting the importance of supply-side supporting policies in the design and implementation of large-scale insurance programs in LMICs.

Our work also contributes to an emerging literature seeking to examine the relationship between health insurance and medical providers’ behaviors, including pharmaceutical innovation in both developed [38, 39] and developing countries [40], physician labor supply [25, 41], and healthcare investment such as inpatient beds and medical equipment [24, 25, 42, 43]. These studies regard supply-side responses as a downside of health insurance expansion because it could induce more healthcare expenses. In the context of developing countries constrained by both the quantity and quality of healthcare resources, supply-side responses could be an important mechanism for the success of their insurance expansion programs. We extend this literature by focusing on China’s NCMS and exploring how much of the supply-side responses explains the success of the program.

## Institutional background

### The New Cooperative Medical Scheme

**Fig. 1:**
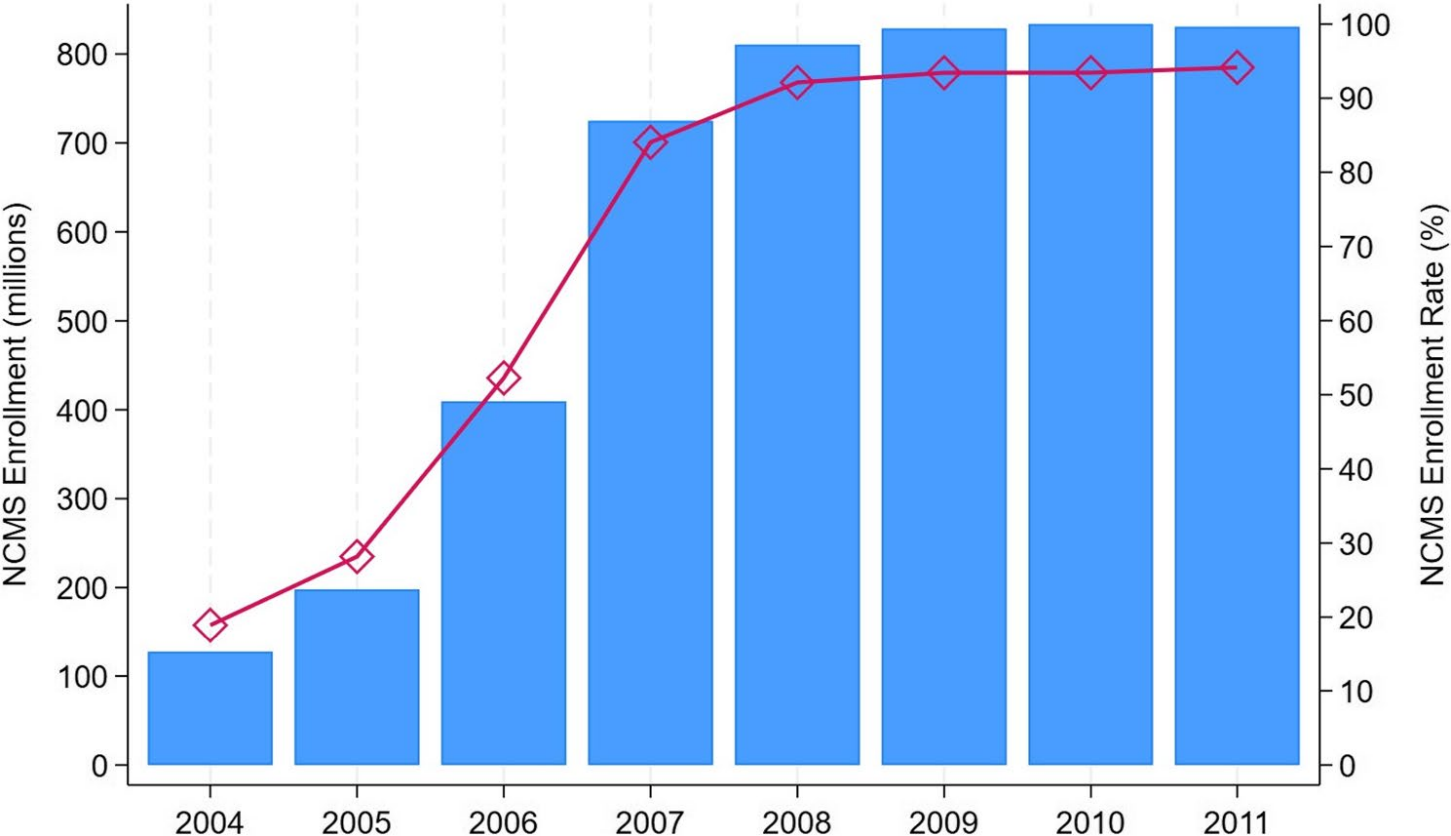
NCMS Enrollment Over Time. Notes: The data source is the NCMS development report by [45]. The y-axis on the left is the number of enrollees. The y-axis on the right is the enrollment rate, which is calculated by dividing the rural population by the number of enrollees over the 2004–2011 period

The NCMS stands as a landmark in China’s health insurance landscape, designed to extend coverage to 640 million rural residents previously uninsured. In 2003, China launched the NCMS program, which was administered independently across approximately 2,800 counties. Under broad guidelines established by the central government, two to three pilot counties were selected in each province during the first year, with additional counties gradually included, aiming for nationwide coverage by 2010. In its inaugural year, only 11% of counties implemented the program. However, its expansion was rapid, and by 2008, nearly all counties had enrolled [44]. As shown in Fig. 1, the program quickly approached full coverage by 2010, with enrollment rising from 18% in 2004 to nearly universal coverage by 2010.

NCMS’s successful expansion can be attributed to two primary factors. Primarily, the government heavily subsidized rural residents’ insurance premiums, where participants contributed merely a fraction of the total—beginning at 10 RMB in 2004, with governmental support covering the remainder. This contribution requirement incrementally rose over the years, as mandated by the central government, leading to a contribution of 10 RMB (0.28%) in 2006, 20 RMB (0.48%) in 2007, 30 RMB (0.63%) in 2008, 50 RMB (0.97%) in 2009, 60 RMB (1.01%) in 2010, and 80 RMB (1.15%) in 2011, with the figures in parentheses representing the percentage of per capita annual income of rural residents in the corresponding year. Additionally, vigorous administrative efforts and promotional activities by the government played a significant role, linking local government budget transfers and officials’ promotions to achieving predetermined enrollment targets [27]. Anecdotal accounts suggest village leaders directly engaged with households to facilitate their enrollment.

However, the implementation of the NCMS, staggered across counties in different years, inherently selected economically more advantageous counties for early adoption, raising potential endogeneity concerns. Related research addressed this issue, finding minimal evidence of economic disparities between provinces with different enrollment gains [27]. Section 5 further investigates whether these minor economic differences influence healthcare demand and supply, with findings indicating no significant impact.

The NCMS system implements differentiated reimbursement policies for local and cross-region care. For local treatment, patients receive direct settlement at designated medical facilities within their prefecture-level cities. Patients are responsible only for out-of-pocket expenses (deductibles and co-insurance) at designated hospitals at the point of service, with the remaining costs settled directly between the insurer and the provider. For cross-region care outside the prefecture-level city, prior registration is required for direct payment; unregistered cases face 10–20% lower reimbursement rates. For non-designated hospitals or unregistered cross-region care, patients must pay upfront and submit claims for reimbursement afterward. In 2023, registration for cross-city care within provinces was eliminated, allowing direct payment for intra-provincial cross-city medical care at designated hospitals without prior registration.

The reimbursement rates vary significantly between inpatient and outpatient care under China’s NCMS system. The program was initially designed to focus on catastrophic spending related to critical illnesses, resulting in relatively generous reimbursement for inpatient services. In the early years of the NCMS, hospitalized individuals faced a deductible of approximately 200–500 RMB (about 6–15% of the net annual income per capita in 2005) [46; 44]. After meeting the deductible, a coinsurance rate of 30–50% applied, with an overall ceiling of 15,000 RMB (about 500% of net annual income per capita in 2005) [47]. Over time, NCMS benefits for inpatient services became more generous, with the coinsurance rate declining to 10–30% [46].

Since the program initially prioritized inpatient care, outpatient services were covered in only 25% of counties [32], with all providers covering inpatient services. Coverage was only limited to outpatient services for certain critical illnesses, such as cancer, organ transplant anti-rejection medications, dialysis for end-stage renal disease, severe mental disorders requiring long-term medication, and antiviral therapies for chronic hepatitis B and C, with reimbursement rates typically ranging from 50 to 70%. Over time, outpatient reimbursement became more generous, expanding to cover a broader range of outpatient expenses.

To mitigate medical costs, the NCMS employs a tiered reimbursement model favoring care from lower-tier providers with more substantial benefits and reducing reimbursements for services from higher-tier facilities. In 2011, primary care providers, including Township Health Centers (THCs) and Community Health Centers (CHCs), received the highest coverage rates (65-90%). County hospitals had the next level of coverage (60-80%), while city hospitals had the lowest (45-70%) [48].

### Healthcare supply in rural China

Rural healthcare in China is structured around a three-tier public system, pivotal for distributing healthcare supply in these regions [31, 49]. At the grassroots, village health clinics, staffed by ”barefoot doctors,” provide basic outpatient care and prescriptions. THCs form the intermediary tier, offering a broader range of inpatient and outpatient services. In more urbanized rural areas, such as in Zhejiang and Jiangsu provinces, CHCs also serve, functioning similarly to THCs but focusing on community residents. County hospitals stand at the apex, delivering the highest quality care compared to the other two tiers.

THCs are crucial in bridging village clinics with county hospitals, facilitating a continuum of care that ranges from preventive services to basic medical care, health education, and family planning [49]. While city hospitals are not officially part of this rural framework, their higher-quality services attract rural residents, particularly those living near urban areas or those with conditions that lower-tier facilities cannot address.^7^ This model underscores the relationship between the three-tier system and healthcare supply, revealing how rural residents navigate China’s healthcare landscape [27].

The healthcare resource landscape in China is shaped by a triad: government entities, public healthcare providers, and private sector participants, with public providers playing a leading role in the healthcare market. Government interventions significantly influence healthcare supply, notably through subsidies directed at public healthcare facilities, particularly in rural regions. A prime example of this dynamic occurred in April 2009, when China initiated a comprehensive healthcare reform aimed at enhancing equitable medical service access for rural populations. This reform period, especially between 2009 and 2011, saw a significant uptick in infrastructure investments targeting primary care facilities in less served areas. Our analysis rigorously incorporates the potential confounding impact of government subsidies through robustness checks, ensuring a nuanced understanding of healthcare supply factors in China.

Since the 1980s, public healthcare providers in China have enjoyed a considerable degree of autonomy in their operations. They have the authority to generate revenue, retain surpluses, and allocate funds, with government subsidies constituting a small and diminishing portion of their income [50]. This shift has motivated public providers to invest in infrastructure and medical equipment, aligning their services with the growing healthcare demands. Moreover, the landscape began to change further in 2000, when private for-profit hospitals were permitted, and private investment in the healthcare sector was actively encouraged. This policy adjustment led to a rapid increase in the number of private medical facilities, which have since played an increasingly significant role in providing outpatient care [51].

China’s public medical providers, including county hospitals, CHCs, and THCs, operate under a semi-autonomous model that grants significant autonomy in financial, managerial, clinical, and investment decisions while maintaining government oversight [52]. Financially, since the early 1980 s, providers have gained increasing autonomy to generate, retain, and manage surpluses, with government subsidies now accounting for just over 10% of THC funding and less than 5% for county hospitals [50]. Public providers can generate revenue through user fees, drug sales, and medical services, and have the autonomy to retain and reinvest surplus income in staffing and allocating funds for infrastructure and technological upgrades, such as expanding capacity or adding new departments, although major capital projects require approval from local health authorities. However, financial autonomy is limited by government-set price controls on medical services and drugs.

In terms of management, public providers can hire and fire staff, set performance targets, and establish internal reward systems, but key leadership appointments remain under the control of local health authorities or government agencies. Clinical autonomy is restricted, as national and provincial health authorities influence medical practice guidelines and treatment protocols. However, financial incentives are prevalent in clinical practices, as the regulated payment schedule incentivizes providers to promote high-tech diagnostics and drugs while neglecting unprofitable basic services.

Additionally, this operational autonomy varies hierarchically among healthcare providers. County-level hospitals, serving as regional medical centers, enjoy greater autonomy in equipment procurement, minor infrastructure projects, and the recruitment of contractual staff compared to THCs and CHCs. In county hospitals, only major projects require administrative approval, whereas even moderate to small equipment purchases in THCs and CHCs require approval.

In China, the establishment of new medical institutions is subject to a rigorous administrative approval process. Health administrative departments, as the competent authorities, are required to develop master plans for the distribution of medical institutions based on regional population, available medical resources, and service demand. Applicants, whether organizations or individuals, must submit their applications to the appropriate health authority. County-level departments are responsible for approving institutions with fewer than 100 beds, while provincial departments oversee the establishment of facilities with 100 or more beds and specialized hospitals. Applicants must provide comprehensive documentation, including an establishment application, a feasibility study report, and site or building plans. Health authorities are required to issue a decision within 30 days of receiving the application and grant a Medical Institution Establishment Approval Certificate to qualified applicants.

**Fig. 2:**
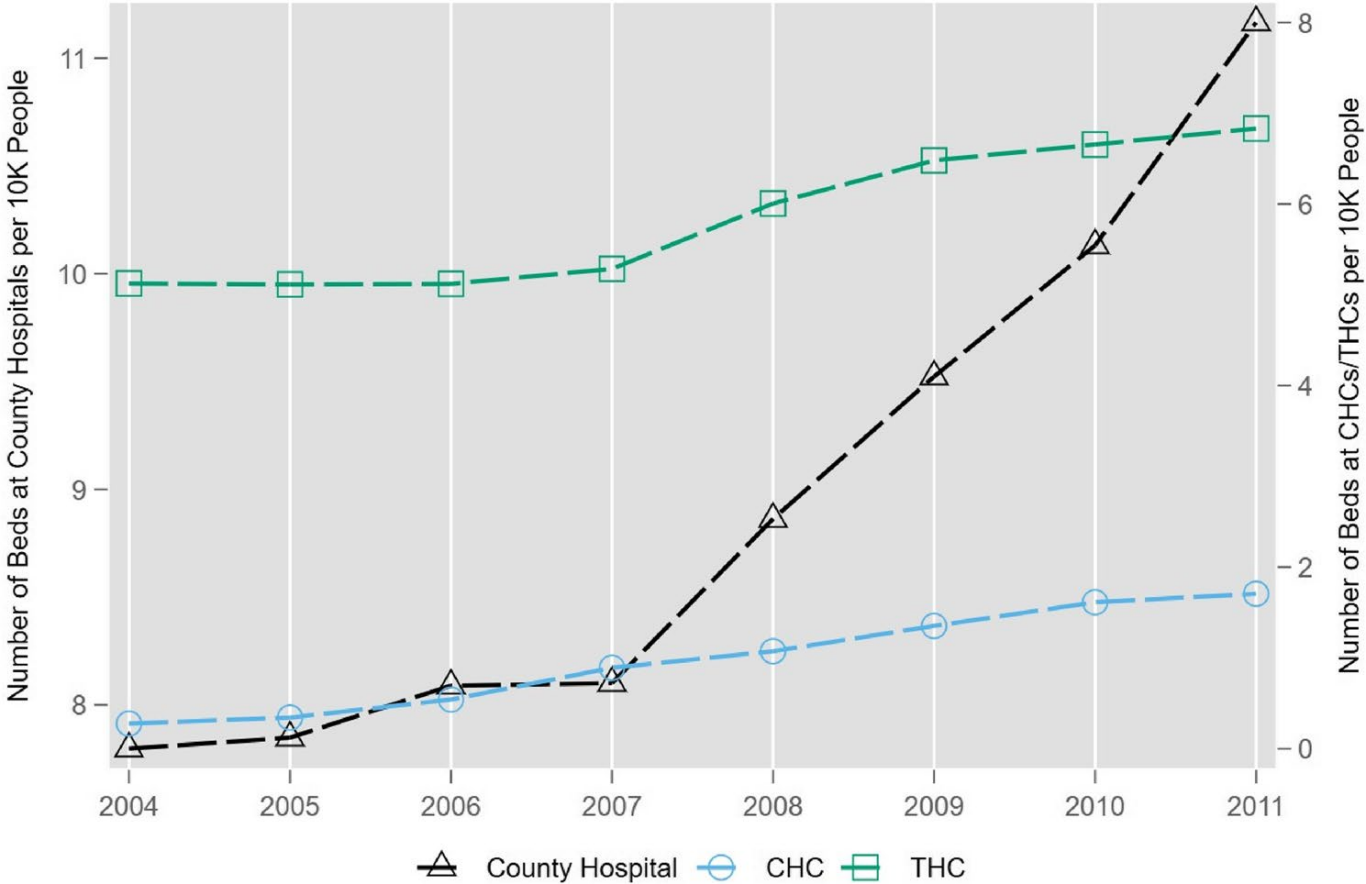
The Number of Hospital Beds Over Time. Notes: The data source is the 2004–2011 CHSY. The y-axis on the left is the number of beds at county hospitals per 10,000 people and the y-axis on the right is the number of beds at CHCs or THCs per 10,000 people over the 2004–2011 period

**Fig. 3:**
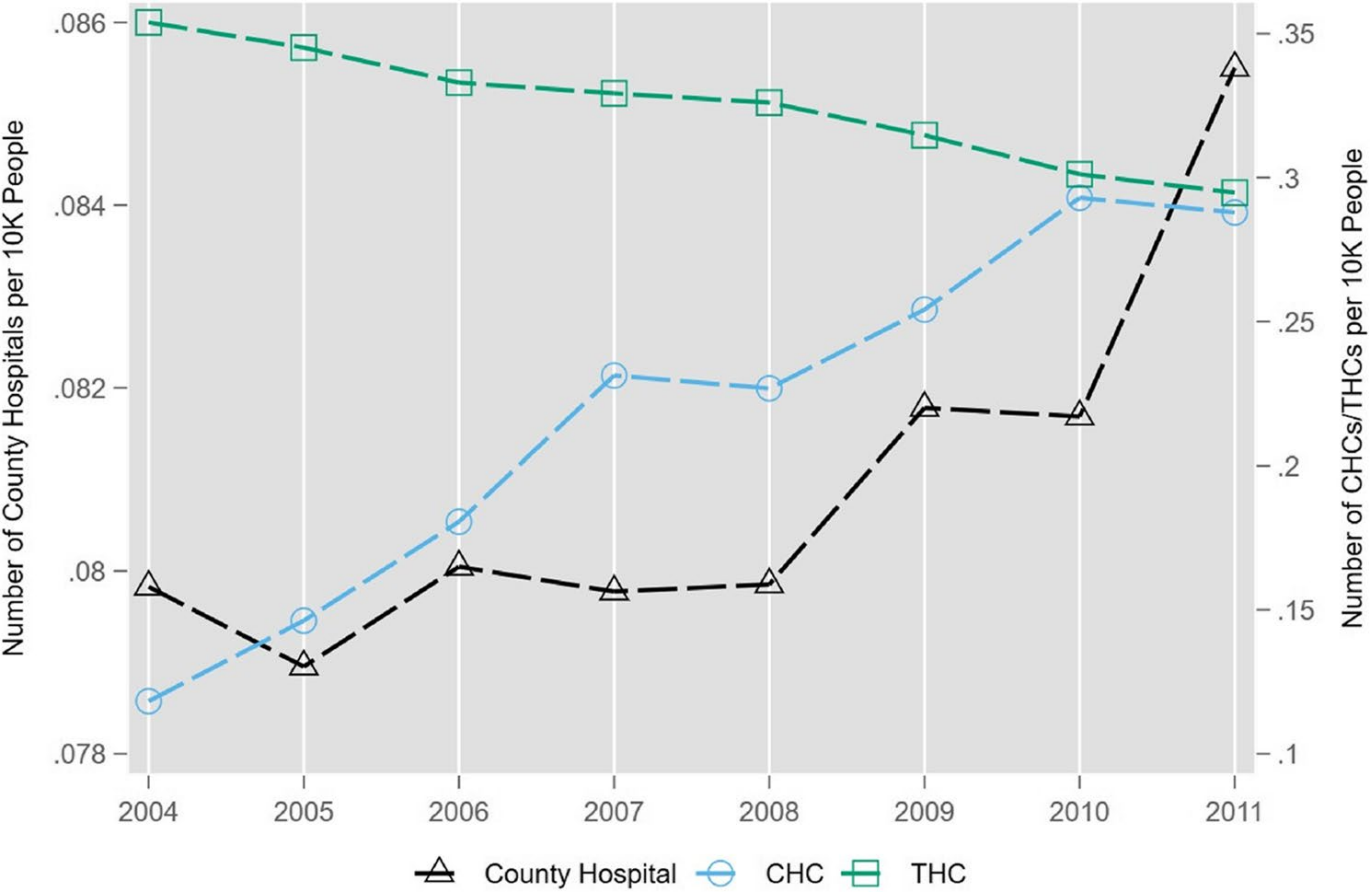
The Number of Medical Institutions Over Time. Notes: The data source is the 2004 to 2011 CHSY. The y-axis on the left is the number of county hospitals per 10,000 people and the y-axis on the right is the number of CHCs or THCs per 10,000 people over the 2004–2011 period

Concurrent with the rollout of the NCMS from 2004 to 2011, rural areas saw a noticeable expansion in medical resources. Specifically, county hospitals experienced a rapid increase in the number of hospital beds, highlighting a significant boost in their capacity to provide care. Meanwhile, CHCs and THCs also observed an uptick in bed numbers, albeit to a lesser extent (as illustrated in Fig. 2). Moreover, the healthcare infrastructure expanded with a considerable rise in the number of CHCs and a notable growth in county hospitals, particularly from 2010 onwards. Contrarily, the quantity of THCs saw a decline over the same period, reflecting a shift in the distribution of healthcare facilities (referenced in Fig. 3). Overall, the quantity of the medical resources in rural areas have improved as a result of the NCMS expansion.

## Data

### Key variables

To examine the responses of supply-side resources to NCMS enrollment expansion in China, we utilize various data sources that provide annual information for each Chinese province. Our analysis is primarily based on data from the China Health Statistical Yearbook (CHSY) for the years 2004 to 2011. Published annually by China’s Health Department, the CHSY offers comprehensive health-related data for each province, including specifics on healthcare resources. In this study, we focus on two main types of healthcare resources: the availability of hospital beds and the number of healthcare providers. We assess these resources by calculating the number of hospital beds and healthcare institutions per 10,000 people in each province. Further, to understand the varied impacts of NCMS enrollment on healthcare supply among different provider levels within the rural three-tier medical system, we classify both hospital beds and healthcare provider counts according to the facility type, including city hospitals, county hospitals, CHCs, and THCs.

Our data on the NCMS policy come from two main sources: the annual China Health Statistical Yearbook (CHSY) for 2007–2011 and the report on NCMS development by [45] for 2004–2006. The CHSY provides NCMS enrollment data, while the report includes information on NCMS enrollment, beneficiaries, and inpatient care reimbursements through graphs for each province. While some years have explicit values reported, others are presented only in graphical form. For years with missing values, we impute the data by using the known values from other years in the report. For instance, the report specifies NCMS enrollment figures for Beijing in 2004 and 2006, but the 2005 value is shown only as a plotted point on a scatter graph. We estimate the 2005 value by referencing the y-axis scale and using software tools to calculate the enrollment proportionally.^8^The proportion of imputed data using the software varies across provinces and years. In 2004, data for 13 out of 31 provinces were imputed; in 2005, 25 provinces; and in 2006, 24 provinces. To assess the accuracy of the imputation, we compare the extracted values with the reported figures for 2011, a year in which raw enrollment rates are available in both the CHSY and the graph presented in [45]. The comparison reveals negligible differences (see Appendix Table A1), suggesting that measurement error introduced by the imputation approach is not a big concern.

The primary independent variable of interest is the NCMS enrollment rate, defined as the ratio of NCMS enrollment to the rural population in each province from 2004 to 2011. Additionally, to ensure a comprehensive examination, we incorporate data on demographic and economic conditions from the annual China Statistical Yearbook (CSY). These variables are used as crucial controls in our model.

### Sample statistics

Table A2 presents the summary statistics for the key variables discussed in our study. Initially, in 2004, NCMS enrollment rate was at 19%, which averaged approximately 70% throughout the 2004–2011 timeframe. In terms of healthcare resource distribution, city hospitals have the highest allocation, with 16 inpatient beds per 10,000 people. In comparison, county hospitals, CHCs, and THCs provide 9, 1, and 6 inpatient beds per 10,000 people, respectively. The average per capita availability of healthcare institutions reveals 0.1 urban hospitals, 0.08 county hospitals, 0.22 CHCs, and 0.32 THCs for every 10,000 individuals. Regarding healthcare utilization, the data indicates that the average number of claims filed per rural resident under the NCMS is about one, suggesting that the typical enrollee accesses NCMS services at least once within the study period. Additionally, rural residents on average visit a doctor twice annually, with roughly 8% utilizing inpatient services.

## Estimation model

In this study, we implement a Two-Way Fixed Effect (TWFE) model, integrating continuous NCMS enrollment rates to explore the responses of healthcare supply to the insurance scheme.^9^The exogeneity of the NCMS enrollment rate is examined by our another related work [27], demonstrating minimal pre-existing trends in NCMS development and finding a negligible correlation between the insured rate in 2004 and both the pre-NCMS levels and trends of healthcare provision and utilization. Leveraging the seemingly exogenous introduction of health insurance for rural populations, the model captures both the intra- and inter-provincial variations in NCMS enrollment rates over time. To this end, we estimate the relationship between the NCMS enrollment rate and healthcare supply using the regression equation specified below:

1 $$\ln\left(Y_{pt}\right)=\beta_0+{\delta NCMS}_{pt}+\eta_P+\mu_{rt}+X_{pt}^{\prime}\beta+\epsilon_{pt}$$

where *Y*_*pt*_ denotes the healthcare resources (e.g. inpatient beds, number of healthcare institutions) available in province *p* in year *t*, normalized per 10,000 people. The outcome variable is logarithmically transformed unless stated otherwise. *NCMS*_*pt*_ represents the continuous NCMS enrollment rate in province *p* during year *t*. The parameter of interest, *δ*, quantifies the relationship between comprehensive NCMS expansion and the targeted outcomes. To account for unobservable factors that are constant over time within each province but may influence both the NCMS rollout and the outcomes of interest, we incorporate province fixed effects *η*_*p*_. These effects control for variables such as governmental enforcement capability, unique health behaviors, and health outcomes. The region-by-year fixed effect *µ*_*rt*_ enables the comparison of provinces within the same region by mitigating the effects of regional shocks or trends that could correlate with the NCMS deployment [27, 54, 55].^10^*X*_*pt*_ incorporates a set of demographic and economic indicators at the province level, encompassing metrics such as population size,^11^ age distribution, educational attainment, marriage rates, gender ratio, and the dependency ratio within households. Economic variables include the unemployment rate, GDP per capita (denominated in 2014 RMB), disposable income levels in rural and urban sectors (also in 2014 RMB), alongside urban consumption patterns and healthcare expenditures (in 2014 RMB). Given that each province consists of urban and rural areas, and since the NCMS targets rural populations, we control for urban consumption and medical expenses to mitigate the influence of healthcare demand from untargeted urban populations. The error term, *ϵ*_*pt*_, captures unobserved factors at the province-year level that may affect healthcare supply. Standard errors are clustered at the provincial level to account for intra-provincial correlation. The weighting of all regression analyses is based on the size of the rural population in the year 2003, ensuring that the study accurately reflects the demographic emphasis of the NCMS program [27].^12^

## Results

In this section, we delve into two interconnected questions: firstly, we examine if the demand shocks generated by the NCMS incentivize medical institutions to enhance their supply capabilities; secondly, we investigate to what extent supply-side changes observed during NCMS implementation are associated with increased healthcare utilization among rural populations.

### Supply-side responses to NCMS

To quantify the relationship between NCMS enrollment rate and healthcare supply in rural areas, we use our baseline model in Eq. (1), and specifically examine two key indicators of healthcare resources among rural providers: the total number of healthcare facilities and the quantity of inpatient beds per 10,000 people. Rural providers refer to county hospitals, CHCs, and THCs, which primarily serve the rural population.

The findings, as detailed in Table 1, illustrate a positive relationship between NCMS enrollment rate and the quantity of rural healthcare infrastructure. Since dependent variables in our analysis are expressed in logarithmic terms, all estimated coefficients, unless otherwise specified, are transformed using the formula *e*^*estimate*^ −1 to obtain a percentage interpretation. An increase in NCMS enrollment rate by 1% point is associated with a 0.14% (0.13 log percent) and 0.06% (0.06 log percent) rise in the number of county hospitals and THCs as presented in columns 1 and 3 of Panel A, respectively, although the increases lack statistical significance. Moreover, there is a statistically significant 0.33% (0.28 log percent) increase in the availability of inpatient beds at THCs (column 3 of Panel B). Given that the NCMS has increased insurance coverage among rural residents by approximately 80% points, and a 1% point increase in NCMS enrollment is associated with a 0.33% rise in inpatient beds at THCs (computed using the formula *e*^0.28^ −1), we can estimate that the overall increase in inpatient bed availability at THCs following the NCMS is about 26% (0.0033 × 80).^13^ Given the mean of 5.28 beds per 10,000 individuals in THCs, the implementation of the NCMS is associated with an additional 1.37 beds per 10,000 individuals (5.28 × 0.26). Overall, our finding indicates that the NCMS is positively correlated with the development of rural healthcare facilities and resources.

**Table 1:** Effects of the NCMS on Healthcare Investments by Rural Medical Providers

	1	2	3
	County Hospital	CHC	THC
	Panel A. Number of Healthcare Providers		
NCMS rate	0.130	−0.108	0.062
	(0.095)	(0.280)	(0.074)
Mean	0.067	0.105	0.334
Observations	231	231	225
Adjusted R-squared	0.963	0.879	0.970
Within R-squared	0.286	0.120	0.197
F-statistic	5.608	8.027	6.060
	Panel B. Number of Beds at Hospitals		
NCMS rate	0.029	1.046	0.282**
	(0.043)	(0.941)	(0.109)
Mean	7.335	0.098	5.280
Observations	231	220	225
Adjusted R-squared	0.977	0.862	0.892
Within R-squared	0.294	0.373	0.379
F-statistic	13.62	15.62	11.46

Each cell reports estimates from the baseline specification (1), with full controls of both time-varying demographic covariates and economic covariates, province fixed effects, and region by year fixed effects on dependent variables per 10,000 people in logarithm form in each panel. CHC denotes community health centers, and THC denotes township health centers. County hospitals and THCs mainly serve rural people, while CHCs are mainly used by urban residents. The mean of each dependent variable is the average in 2004 per 10,000 people and is weighted by the rural population in 2003. All estimates are weighted by the rural population in 2003. Standard errors are clustered by province and are shown in parentheses

***p *<* 0.01, **p< 0.05, *p< 0.10

Our finding that the NCMS is associated with increased investments in patient bed by THCs suggests that the insurance expansion created strong demand-side pressure, encouraging healthcare providers to scale up capacity. This response may reflect both increased patient volumes due to greater financial access and the profit incentives tied to inpatient care under the NCMS reimbursement scheme. The expansion of bed capacity could improve healthcare access and reduce congestion, ultimately enhancing the overall efficiency of rural healthcare delivery. These results underscore the importance of aligning financial incentives with healthcare infrastructure development to ensure that increased demand translates into improved health outcomes. Refer to the discussion section for more details.

Interestingly, the analysis suggests a redistribution effect, where urban primary healthcare providers, such as CHCs, witness a marginal decline of about 0.1% in their numbers, although these findings do not achieve statistical significance. This pattern may reflect a strategic allocation of health resources favoring rural over urban areas within the context of the NCMS. The distinct impact observed in the expansion of inpatient beds, as opposed to the insignificant growth in healthcare facilities, could be attributed to the lower costs associated with augmenting existing infrastructure (e.g., adding beds) compared to the substantial fixed expenses required for establishing new healthcare centers or hospitals. In conclusion, our findings underscore the pivotal role of the NCMS in expanding critical healthcare infrastructure by increasing inpatient bed capacity.

The supply-side adjustments following the expansion of the NCMS in China are comparable in magnitude to those observed in more advanced healthcare systems. Our analysis indicates a significant 0.33% increase in the number of inpatient beds at THCs with the NCMS program in implementation, although the bed capacity in county hospitals remained largely unchanged. Given that inpatient beds in THCs accounts for about 40% of total inpatient beds in rural healthcare providers, we can infer an average increase of about 10% in rural inpatient bed availability when extrapolated to a full (0–100%) expansion in coverage. This increment mirrors findings from other countries with advanced healthcare systems. Specifically, research conducted in Japan identifies a comparable 10% rise in inpatient bed capacity following health insurance expansions [24]. Similarly, an increase of 20–30% in medical technology adoption post-expansion of health insurance coverage was found in the United States [5]. The supply responses reported in both studies reflect the contrast between full (100%) and no insurance coverage, making them directly comparable to our estimates above. While we lack direct data to compare the expansion of medical technology in China with that of the United States, these findings suggest a noteworthy alignment of China’s supply-side responses with international experiences.

Even though the quantitative impact on medical technology in China remains unmeasured due to data limitations, the increase in inpatient beds within rural healthcare facilities post-NCMS implementation closely aligns with the upper bounds of the response seen in the United States and directly parallels the outcome observed in Japan. Thus, without necessarily exceeding the supply-side enhancements seen in the U.S., China’s performance in expanding healthcare resources post-NCMS implementation matches or mirrors the experiences of developed nations like Japan, serving as a significant benchmark in the global context of healthcare system responses to insurance expansion.

### Supply-side mediation of NCMS efficacy

To further investigate whether supply-side responses to NCMS enrollment can translate into improved healthcare utilization, we examine the extent to which the increased medical resources can mediate the relationship between NCMS enrollment and health services use. Our analysis concentrates on two critical types of healthcare: inpatient care and outpatient care, which have been shown to increase significantly following the NCMS, as documented by the closely related study by [27]. We then assess the contribution of supply-side responses to the NCMS’s efficacy through exploring their potential roles as mediators in the relationship between NCMS enrollment and outpatient and inpatient care use. Should the increased supply substantially influence the NCMS’s impact on healthcare utilization, we anticipate these supply responses to be associated with a meaningful share of the observed increase in healthcare utilization among rural residents. Such an analysis not only clarifies the importance of supply-side responses but also highlights their mediating effects, providing insights that complement and build upon the findings in [27].

Panel A of Table 2 presents the effects of NCMS on inpatient stays in healthcare providers serving rural residents (CHCs, county hospitals, and THCs). NCMS enrollment is positively associated with inpatient utilization at these facilities, consistent with [27]. Among rural providers, the hierarchical reimbursement scheme under NCMS creates stronger incentives for rural residents to seek inpatient care from primary healthcare providers (CHCs and THCs). The substantial percentage increase in inpatient stays in CHCs can be explained by the low baseline utilization rate of CHCs for inpatient care—0.99 per 10,000—compared to 169.1 per 10,000 in county hospitals and 127.6 per 10,000 in THCs.

**Table 2:** The effect of NCMS enrollment on inpatient care use controlling for rural healthcare resources

	(1)	(2)		(3)	(4)
	Total	County Hospital		CHC	THC
	Panel A. Baseline Estimates				
NCMS rate	0.114*	0.116***		1.647*	0.360*
	(0.065)	(0.041)		(0.850)	(0.184)
Mean	479.4	169.1		0.99	127.6
Adjusted R-squared	0.970	0.983		0.862	0.928
Within R-squared	0.182	0.235		0.267	0.408
F-statistic	16.11	19.09		12.39	7.237
	Panel B. Number of Beds at Hospitals				
NCMS rate	0.052		0.070**	0.892**	−0.023
	(0.057)		(0.026)	(0.414)	(0.124)
THC beds	0.242		0.086	−0.020	1.162***
	(0.143)		(0.056)	(0.495)	(0.135)
County hospital beds	0.343**		0.759***	0.120	0.324
	(0.150)		(0.077)	(0.840)	(0.272)
CHC beds	−0.005		−0.007	1.050***	0.003
	(0.010)		(0.005)	(0.127)	(0.021)
Observations	210		214	210	214
Adjusted R-squared	0.977		0.993	0.941	0.961
Within R-squared	0.379		0.695	0.691	0.679
F-statistic	22.51		63.28	59.26	179.2
	Panel C. Number of Healthcare Providers				
NCMS rate	0.096		0.075*	1.767**	0.278
	(0.066)		(0.043)	(0.716)	(0.178)
Number of THCs	0.137		0.125	0.720	1.283***
	(0.118)		(0.098)	(0.607)	(0.165)
Number of county hospitals	0.081		0.326***	−0.116	0.076
	(0.128)		(0.077)	(0.876)	(0.221)
Number of CHCs	0.021		0.002	0.645**	0.060
	(0.022)		(0.014)	(0.314)	(0.054)
Observations	210		225	210	225
Adjusted R-squared	0.970		0.986	0.870	0.950
Within R-squared	0.197		0.405	0.319	0.592
F-statistic	33.08		43.61	21.31	84.66

Each cell reports estimates of the effects of NCMS enrollment on inpatient care use after controlling for each set of healthcare resources: hospital beds and number of providers in rural areas using the baseline model (1) with full controls of both time-varying demographic covariates and economic covariates for each province, province fixed effects, and region-by-year fixed effects. CHC denotes community health centers, and THC denotes township health centers. Each column corresponds to the estimates of inpatient care at specific hospitals. The mean of the dependent variable is the average of inpatient care use in 2004 per 10,000 people and is weighted by the rural population in 2003

***p< 0.01, **p *<* 0.05, *p *<* 0.10

Panel B and C of Table 2 report the effects of NCMS enrollment on inpatient care utilization after controlling for the number of inpatient beds (Panel B) and healthcare institutions (Panel C) among rural healthcare providers. Panel B shows that controlling for hospital beds decreases the baseline estimate of the relationship between NCMS enrollment and total inpatient stays by 6% points (0.30 inpatient stays per 10,000 people), by 75% points (0.07 inpatient stays per 10,000 people) at CHCs, by 5% points (0.08 inpatient stays per 10,000 people) at county hospitals, and by 38% points (0.49 inpatient stays per 10,000 people) at THCs, compared to the baseline estimates. It is noteworthy that the effect of NCMS enrollment on inpatient services utilization at THCs is close to zero after controlling for inpatient beds (Column 4), which suggests that the baseline effect at THCs is mainly driven by increased bed capacity following the NCMS. Panel C shows further evidence that the number of providers also plays a role in the relationship between NCMS enrollment and inpatient care use, but smaller than the role of inpatient beds, which is reasonable given that the number of inpatient beds directly impacts the capacity for inpatient services. Appendix Table A3 reports the estimates of the relationship between NCMS enrollment and inpatient use after simultaneously controlling for all rural healthcare resources (both the number of healthcare providers and hospital beds). Compared to the baseline estimates, responses from medical resources account for about 65% of NCMS enrollment’s effect on total inpatient services utilization, 27% of NCMS enrollment’s effects at CHCs, 32% of NCMS enrollment’s effects at county hospitals, and absorb all of NCMS enrollment’s effects at THCs.

Panel A of Table 3 reports the estimated effects of NCMS enrollment on outpatient visits to healthcare providers serving rural residents (CHCs, county hospitals, and THCs). Since NCMS primarily covers inpatient services, it is not surprising that NCMS enrollment does not significantly affect outpatient visits to any of these providers. Given the significant increase in inpatient bed capacity at THCs, we focus on how the expansion of medical resources mediates outpatient visits to THCs. The results in column 4 of Panel B of Table 3 align with our expectations: the increase in inpatient beds at THCs does not significantly affect outpatient visits to THCs by rural residents. After controlling for medical resources, the baseline effect’s magnitude decreases substantially, though it remains statistically insignificant. This provides weak evidence that the increase in outpatient visits to THCs (if any) following NCMS implementation may be partially correlated with improvements in rural healthcare supply.

To conclude, column 4 of Table 2 shows that the supply-side responses in healthcare resources account for about half of the baseline effects of NCMS enrollment on inpatient visits at THCs. The NCMS enrollment not only increases the quantity but also plausibly enhances the quality of medical resources, which works with the NCMS insurance expansion to increase rural people’s healthcare use.

**Table 3:** The effect of NCMS enrollment on outpatient visits controlling for rural healthcare resources

	(1)	(2)		(3)	(4)
	Total	County hospital		CHC	THC
	Panel A. Baseline Estimates				
NCMS rate	−0.060	0.077		−0.076	0.070
	(0.050)	(0.056)		(0.433)	(0.149)
Mean	15,293	3,599		602.4	5,365
Adjusted R-squared	0.991	0.981		0.912	0.906
Within R-squared	0.164	0.317		0.0348	0.191
F-statistic	5.242	35.37		1.035	1.459
	Panel B. Number of Beds at Hospitals				
NCMS rate	−0.086*		0.060	−0.395	−0.001
	(0.044)		(0.043)	(0.365)	(0.170)
THC beds	−0.009		−0.059	0.190	0.411
	(0.041)		(0.042)	(0.261)	(0.244)
County hospital beds	0.194**		0.666***	−1.168*	−0.137
	(0.082)		(0.073)	(0.581)	(0.298)
CHC beds	0.005		0.001	0.190**	−0.009
	(0.008)		(0.007)	(0.072)	(0.022)
Observations	214		214	214	214
Adjusted R-squared	0.993		0.991	0.926	0.914
Within R-squared	0.269		0.659	0.229	0.269
F-statistic	10.13		214.8	8.862	2.444
	Panel C. Number of Healthcare Providers				
NCMS rate	−0.071		0.022	0.116	0.051
	(0.044)		(0.039)	(0.312)	(0.159)
Number of THCs	0.051		0.013	0.003	0.654***
	(0.046)		(0.051)	(0.240)	(0.227)
Number of county hospitals	0.107*		0.419***	−0.644	−0.181
	(0.056)		(0.062)	(0.379)	(0.191)
Number of CHCs	0.032**		−0.009	0.704***	−0.029
	(0.012)		(0.016)	(0.104)	(0.024)
Observations	225		225	225	225
Adjusted R-squared	0.991		0.989	0.947	0.922
Within R-squared	0.232		0.592	0.442	0.331
F-statistic	4.077		36.45	9.960	4.077

Each cell reports estimates of NCMS enrollment’s on outpatient visits after controlling for each set of healthcare resources: hospital beds and number of providers in rural areas using the baseline model (1) with full controls of both time-varying demographic covariates and economic covariates for each province, province fixed effects, and region-by-year fixed effects. CHC denotes community health centers, and THC denotes township health centers. The mean of the dependent variable is the average of outpatient visits in 2004 per 10,000 people and is weighted by the rural population in 2003. All estimates are weighted by the rural population in 2003. Standard errors are clustered by province and are shown in parentheses

***p *<* 0.01, **p *<* 0.05, *p *<* 0.10

### Robustness checks

#### Concurrent policies

Concerns may arise that the observed supply-side responses are influenced not solely by NCMS enrollment but also by concurrent government initiatives aimed at bolstering rural healthcare infrastructure. In April 2009, China’s central government officially launched a systematic fiscal subsidy program for primary healthcare institutions, covering 70–90% of personnel expenses and providing subsidies for medical equipment purchases based on criterion such as staff size and performance, with priority given to central and western regions and impoverished areas. Additionally, it allocated funds for essential public health services based on population size. The policy was rolled out in phases, with pilot regions receiving funds by July 2009 and nationwide coverage achieved by the first quarter of 2010. The supply-side medical reform significantly augmented subsidies for medical providers and investments in rural healthcare facilities [28]. This overlap raises concerns about the attributability of the improvements in healthcare supply directly to NCMS enrollment. To address these concerns and disentangle the effects of NCMS enrollment from those of the 2009 medical reform, we employ an event study model [27].

2 $$ln\left(Y_{pt}\right)=\alpha_0+{NCMS}_p\times\left[\sum_{y=1}^7\theta_y1\left\{t-2004=y\right\}\right]+\eta_p+\mu_{rt}+X_{pt}^{\prime}\beta+\in_{pt}$$

where *NCMS*_*p*_ indicates NCMS enrollment rate in province *p* for the year 2004. The model utilizes event-year dummies, 1{*t* − 2004 = *y*}, which take the value one for each observed year *t* = 2005,2006,*…*,2010,2011, respectively, comparing these years against the baseline year of 2004, which is excluded as the reference year in the analysis. The rest of the variables and specifications adhere to those presented in the baseline Eq. (1).

**Fig. 4:**
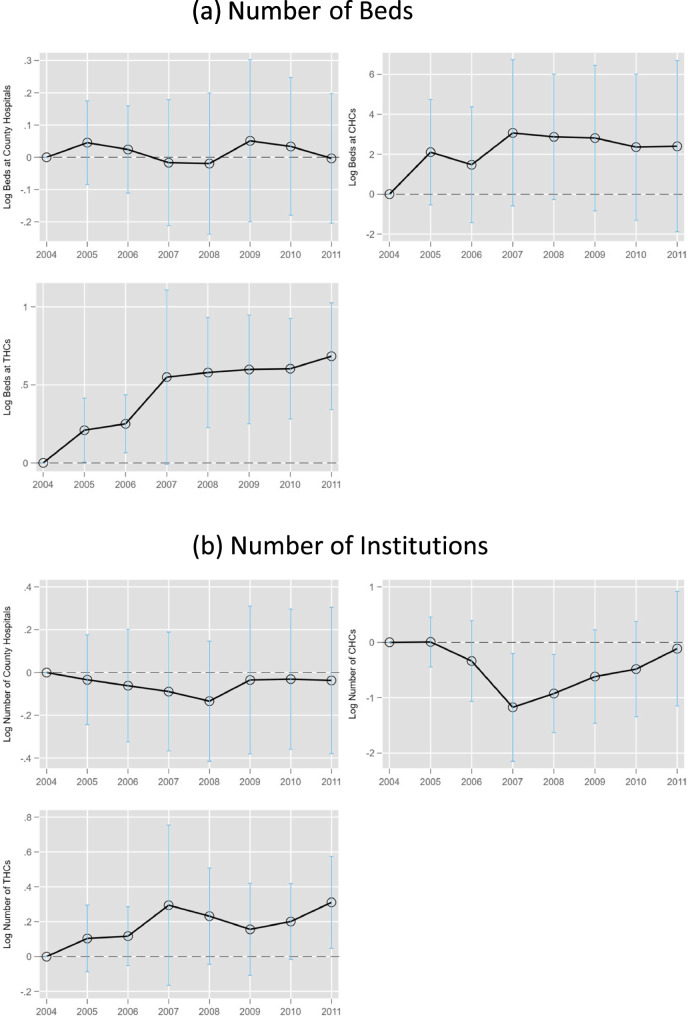
Event-Study Estimates by NCMS Enrollment Gain. Notes: The data source is the 2004–2011 CSY. Each figure plots the event-study estimates in specification (2). The y-axis is the dependent variable in log form. The interval is the 95% confidence interval of each estimate

Figure 4 illustrates the relationship between NCMS enrollment and the availability of healthcare resources in rural areas using Eq. (2). Should the escalation of government investments post-2009 be the primary catalyst for supply-side adaptations, a pronounced enhancement post-2009 would be anticipated. However, in contradiction to such expectations, Fig. 4 reveals no sudden surge in supply-side effects subsequent to 2009. Instead, it indicates that the supply responses commenced prior to 2009, challenging the concern that these adaptations are exclusively a result of the autonomous medical reform launched in 2009.

As shown in Fig. 4(b), the NCMS insurance expansion os associated with a decline in the number of CHCs but an increase in the number of THCs in 2007. This substitution reflects a strategic shift in healthcare provision, reallocating more resources to THCs, which primarily serve rural residents. Specifically, to enhance efficiency in CHCs, starting in 2008, the government merged some CHCs—eliminating weaker-performing ones and consolidating them with betterfunctioning facilities. Despite the reduction in the number of CHCs, healthcare capacity within CHCs increased, as shown in Fig. 4(a), where the number of inpatient beds in CHCs rose sharply in 2007. However, it is important to note that our baseline findings in Table 1 show no statistically significant effects on the number of medical institutions.

Our main results are also stable when only using data from 2004 to 2008 when there was few confounding healthcare reforms, as shown in Appendix Table A4 and A5.

#### Additional outcomes with respect to quality of care

To capture the quality effects of the insurance expansion, rather than just the quantity, we complement the analysis by considering alternative outcomes as shown in Appendix Table A6. This analysis suggests that the increase in NCMS enrollment significantly reduces the incidence rate of infectious diseases (per 100,000 individuals). Importantly, this reduction appears to be primarily driven by the increased use of both inpatient and outpatient services by rural residents, which is made possible by the expansion of insurance coverage. Additionally, the increased utilization of healthcare services among rural populations has likely improved the early detection and treatment of infectious diseases, contributing to the observed decrease in incidence rates. We did not find significant evidence regarding other types of mortality, which we believe is reasonable, as infectious diseases are more easily controlled when individuals have better access to the healthcare system, compared to other conditions.

#### Heterogeneous supply responses

To show that our supply responses as revealed in Table 1 are indeed generated by the NCMS expansion, we complement it with two additional heterogeneous analysis. Firstly, if NCMS enrollment does generate increased demand for healthcare, supply-side responses should be more prominent in areas where healthcare resources were relatively scarce prior to the policy rollout. Therefore, we conduct a heterogeneity analysis based on China’s three major regions—western, middle, and eastern—which are known to differ significantly in baseline healthcare supply conditions. In China, the western region is generally characterized by low pre-existing medical capacity and more pronounced shortages in healthcare infrastructure. In contrast, the eastern region typically enjoys relatively abundant healthcare resources. Based on this gradient, we hypothesize that supply-side responses to NCMS should be stronger in the western and middle regions than in the east.

Appendix Table A7 presents these heterogeneous supply responses across regions by facility type using a split-sample approach. As each regression includes fewer than 100 province-year observations, the findings should be interpreted with caution. The reason is that the smaller sample size may exacerbate potential endogeneity concerns, as there is less variation in NCMS enrollment exploited in each regional subsample, potentially weakening the credibility of identifying variation. Despite these caveats, we still observe meaningful patterns. As our baseline results in Table 1 show that the most robust and significant changes occur in township health centers (THCs), particularly in the number of beds. Therefore, in this heterogeneity analysis, we place greater emphasis on interpreting results for THCs (columns 7–9 of Appendix Table A7), followed by CHCs and county hospitals.

As shown in columns 7–9 of Appendix Table A7, we find the clearest and most consistent evidence supporting our hypothesis. In the western region, THC bed capacity increases substantially, even though the number of institutions remains stable; in the middle region, both the number and size of THCs decline; in the eastern region, there is no statistically significant change in either measure. This pattern strongly aligns with our expectation that supply-side responses are concentrated in resource-scarce settings, the western region in this context, where new demand from NCMS enrollment places more pressure on existing infrastructure.

Conversely, responses for county hospitals show regionally complex dynamics possibly influenced by the fact that they serve both rural and urban populations. In particular, as revealed in columns 1–3 of Appendix Table A7, while the number of county hospitals does not increase significantly in the western region, the number of beds expands considerably, suggesting a shift toward scaling up existing facilities rather than building new ones. In contrast, in the middle and eastern regions, we observe increases in the number of county hospitals, but limited or no change in their bed capacity. These findings appear somewhat counterintuitive, as we would expect the largest expansion to occur in resource-constrained western regions. A potential explanation is that county hospitals often serve both rural and urban populations, especially in the eastern region where urbanization is higher. Therefore, when the endogeneity issue is exacerbated by reduced variation in the data, the observed expansions in the number of county hospitals in more developed regions may be driven in part by urban demand, thereby obscuring the rural-specific supply response pattern we aim to capture.

The heterogeneous responses by CHCs are more aligned with our expectation (columns 4–6 of Appendix Table A7). In the western region, we observe a larger increase in the number of CHCs and a significant increase in CHC bed capacity, though the effect on quantity is statistically insignificant. In the middle region, both the number and size of CHCs appear to decline. In the eastern region, we find no significant change in the number of CHCs, but a surprisingly large increase in bed capacity. These findings show that while CHCs in western regions respond in both number and size, CHCs in the eastern region experience a slight decline in number but a substantial increase in size. Since expanding the number of facilities typically requires greater investment, this pattern suggests that western regions exhibit stronger supply responses than eastern regions.

Our second heterogeneity analysis concerns rural versus urban provinces. As the NCMS was primarily targeted toward rural populations, we would expect stronger supply-side responses in provinces with a higher proportion of rural residents. We classify provinces into rural-dominant and urban-dominant categories based on whether their rural population share is above or below the national median. We then conduct subsample analyses by facility type, with the results reported in Appendix Tables A8. Across county hospitals, CHCs and THCs, we find consistent results that provinces with a larger rural population share experienced stronger healthcare supply responses following NCMS expansion. As shown in columns 1–2, in rural-dominant provinces, we observe a larger increase in the number of county hospitals, and a comparable increase in bed capacity relative to urban-dominant provinces. As shown in columns 3–4, both the number of CHCs and the number of beds increase more substantially in rural provinces than in urban ones. The results in columns 5–6 also show a significantly larger increase in the number of beds in THCs in rural provinces compared to urban ones. While some estimates are not statistically significant—likely due to the smaller sample sizes—these patterns generally support our hypothesis that supply-side changes are more pronounced in areas more directly exposed to the NCMS.

#### More robustness checks

To address concerns about inconsistent sample sizes due to zeros in the outcome variables in Table 2, we conduct robustness checks using both a log-plus-constant transformation (log(y + 0.01)) and the inverse hyperbolic sine transformation. These approaches allow us to retain all 231 province-year observations. The results, presented in Appendix Table A9, are consistent with the main analysis and confirm that the observed effects are not sensitive to data transformation choices. As another robustness check, Appendix Table A10 and A11 show that our baseline and mediation results are consistent without including any controls. A12 shows that our main result remains stable and significant across alternative specifications with sequential inclusion of fixed effects, covariates, and weights.

## Conclusion and discussion

Our analysis evaluates the relationship between NCMS enrollment and healthcare resources in rural China, highlighting the significant role of supply-side responses in mediating the scheme’s effectiveness. The NCMS is a major initiative aimed at expanding insurance coverage for rural populations in LMICs. Using a province-year panel dataset spanning eight years after the NCMS’s nationwide implementation in 2004, we investigate the dynamics of healthcare supply changes following the scheme. Our findings reveal a notable increase in hospital bed availability subsequent to NCMS enrollment, with a minimal change in the number of healthcare institutions. Compared to outcomes in high-income countries, the supply-side responses we identified are significant. Adjusting for variations in healthcare resources, we find that the relationship between NCMS enrollment and inpatient care utilization is halved, emphasizing the crucial role of rural healthcare providers adapting to increased demand for the success of insurance expansion initiatives. This mirrors results found in [24], indicating the universal importance of responsive healthcare supply in the effectiveness of public insurance expansions.

Three key factors drove the expansion of infrastructure in THCs following the NCMS: a significant surge in demand, a tiered reimbursement scheme, and profit-seeking incentives. The NCMS expansion, which primarily covers inpatient services, significantly increased demand for inpatient care by enrolling an additional six hundred million rural beneficiaries. Under the tiered reimbursement scheme, patients receive the highest reimbursement rates for treatment at THCs, incentivizing them to seek care at these facilities rather than higher-level hospitals. Moreover, THCs operated as self-financing facilities, with government subsidies accounting for only 10% of total revenue [56]. As a result, THCs were motivated to generate profits to sustain operations. Medical staff and doctors in THCs also had profit-driven incentives, as their incomes were tied to revenue-based performance indicators. Faced with a sharp increase in demand for inpatient care and aligned profit motives, THCs were fully incentivized to accommodate more patients under the regulated payment structure. Before 2012, hospitals were permitted a 15% markup on drug sales, further encouraging THCs to expand bed capacity to maximize patient volumes and drug-related revenue. Similar economic incentives have played a key role in the supply-side response to insurance expansion in the US, where private medical facilities benefit from increased payment rates and patient volumes [57]. Although China’s regulated payment rates are lower than those in the US, the large patient base creates economies of scale and reduces medical costs, thereby providing sufficient economic incentives for healthcare providers to expand supply.

The study underlines the urgent need to address supply-side constraints and increase investment in healthcare infrastructure as essential policy directions to meet the growing healthcare demands of low-income populations in LMICs. To strengthen supply-side responses in LMICs, several targeted policy measures can be implemented. First, maintaining a sufficiently large market to encourage supply-side investment is essential. This can be achieved by optimizing the tiered reimbursement scheme, setting higher reimbursements for providers with insufficient supply responses, and expanding online medical care to reach underserved, sparsely populated areas. Second, financial incentives for healthcare providers should be strengthened through measures such as increasing reimbursement rates for both inpatient and outpatient services and introducing performance-based funding.

## Supplementary Information

Supplementary Material 1.

## Data Availability

The data and codes that support the findings of this study are available at the following replication link: https://doi.org/10.17605/OSF.IO/4HP83

## Footnotes

1 Nations such as Colombia in 1993, Ghana, Vietnam, and China in 2003, Mexico in 2005, Georgia in 2006, and Nicaragua in 2007 have undertaken substantial insurance expansions.

2 The U.S. public insurance programs, for instance, are shown to raise healthcare utilization and reduce mortality rates, as documented in studies by [1–18].

3 Research in Mexico [19], Nicaragua [20], Georgia [21], and India [22] has highlighted this trend. A thorough review by [23] offers comprehensive insights.

4 Despite successes in expanding coverage, the benefits are more limited in developing countries. In 2014, on average, 36.26% of medical spending in LMICs was out of pocket, compared to only 13.63% in OECD countries. For more details, see the WHO Global Health Expenditure Database at https://apps.who.int/nha/database.

5 Xiaoyan Lei and Wanchuan Lin [30] find that the introduction of the NCMS leads to increases in the number of general physical examinations, but has little effects on the numbers of inpatient stays or outpatient visits, out-of-pocket expenses, or health improvements using the 2000, 2004, and 2006 CHNS data [29], conduct a longitudinal survey in 2002 and 2005, and employ a DID method to show that the NCMS does not increase outpatient visits [31], also estimate a DID model using two waves of survey data for five provinces (Jiangsu, Sichuan, Shaanxi, Jilin, and Hebei) in China in 2005 and 2008, and find little evidence that being enrolled in the NCMS increases the likelihood of visiting healthcare providers when sick.

6 In particular, [32] use a DID design from the National Health Service Survey (NHSS) for 2003 and 2005 covering 12 provinces and show that the NCMS increases inpatient stays and outpatient visits, particularly at township health centers [33], also uses a DID model from the CHNS for 1993 to 2011 showing that the NCMS is effective in insuring households against health shocks, and in helping them invest in their children’s education. The most recent study by Huang [34] exploits the enhancement of insurance benefits since the integration of rural-urban insurance in 2009 in a staggered DID design, and shows that the increased reimbursement rates lead to higher inpatient care utilization by middle-aged and older residents and lower chances of having high blood pressure with suggestive evidence. Of the studies that examine the NCMS’s effects on medical OOP expenditures, some find that it reduces OOP expenses only slightly [30, 32, 35–37], while others show that it reduces them substantially [31]. There are a handful of papers that investigate the health effects of the NCMS [37], use a panel data of the Chinese Longitudinal Healthy Longevity Survey with a DID method and find that the insurance significantly improves cognitive functions for rural elderly while has no effects on other health outcomes such as general health status and mortality rate. Xiaoyan Lei and Wanchuan Lin [30] use the CHNS with individual fixed effects and do not find significant health benefits from the NCMS.

7 Patients with chronic or rare diseases also seek care at city hospitals outside their province, as China’s referral system allows patients to self-refer to any provider post the market reforms of the 1980s.

8 The software tool is called CorelDRAW, a vector-based graphic tool that allows precise measurement based on axis scaling.

9 While the staggered DiD estimator for continuous treatments developed by [53] is promising, this method is primarily designed for settings where treatment intensity changes once per unit (i.e., staggered but discrete shifts). In our case, NCMS coverage varies continuously both across provinces and over time, making the treatment structure incompatible with the assumptions of this estimator. We therefore rely on a continuous two-way fixed effects and region-by-year fixed effects to account for differential time trends and treatment intensity.

10 China’s 31 provinces are grouped into five regions based on geographic location and economic conditions: eastern, northern, middle, southern, and western regions. Provinces within the same region share similarities in geographical features, government policies, and socio-economic statuses.

11 We control for province population to account for potential general equilibrium effects on healthcare supply dynamics and variations in the existing stock of healthcare infrastructure that may not be fully captured by per capita adjustments.

12 We weight each province by the share of its rural population because the NCMS insurance expansion primarily targets rural residents. Weighting by rural population ensures that provinces with larger rural populations, where the policy’s impact is more pronounced, are given greater representation in the analysis, thereby improving the accuracy and relevance of the estimated effects.

13 While the 26% increase in the number of beds per 10,000 inhabitants at THCs may appear large, this substantial percentage increase reflects the initially low inpatient bed density in rural medical facilities before the NCMS reform. Prior to the NCMS, healthcare resources were heavily concentrated in higher-level hospitals, leaving rural facilities relatively under-resourced. Specifically, during our sample period, THCs had only about 5.28 beds per 10,000 inhabitants, and county hospitals had around 7.34 beds per 10,000 inhabitants (see Table 1), resulting in a total inpatient bed density in rural China of just 12.62 beds per 10,000 inhabitants—well below those in high-income countries (126 per 10,000 in Japan and 280 per 10,000 in the US). The small baseline stock of beds at THCs means that even a moderate absolute increase would translate into a large percentage increase, thereby explaining the seemingly high marginal effect.
