# Supplementary material for: The role of supply responses in public insurance expansion: evidence from the New Cooperative Medical Scheme in China
Source: BMC Health Serv Res. 2025 Jul 16;25:955. doi: 10.1186/s12913-025-13090-0 (PMC12269228; doi:10.1186/s12913-025-13090-0)
Supplement: Supplementary file 1 — Supplementary Material 1. [file 12913_2025_13090_MOESM1_ESM.pdf]

Appendices

A Tables and Figures

Table A1: Validation of Extracted NCMS Enrollment Data Against Actual Values for 2011

Province	Year	Raw Data	Predicted Data	Differences
Beijing	2011	276.83	276.5	-0.00119
Hebei	2011	5020.02	5002.32	-0.00353
Shanxi	2011	2194.4	2193.2	-0.00055
Heilongjiang	2011	1418.8	1416.5	-0.00162
Jiangsu	2011	4373.91	4372.6	-0.0003
Anhui	2011	4917.1	4923.4	0.001281
Fujian	2011	2441	2440.66	-0.00014
Jiangxi	2011	3240.5	3243.2	0.000833
Shandong	2011	6629.13	6667.18	0.00574
Henan	2011	7804.46	7806.53	0.000265
Hunan	2011	4654.96	4650.6	-0.00094
Guangdong	2011	2849.93	2847.56	-0.00083
Guangxi	2011	3953.47	3957.68	0.001065
Hainan	2011	485.46	484.35	-0.00229
Chongqing	2011	2224.37	2226.56	0.000985
Guizhou	2011	3074	3071	-0.00098
Gansu	2011	1918.27	1923.69	0.002825
Qinghai	2011	347.87	346.41	-0.0042
Ningxia	2011	370.25	369.59	-0.00178
Xinjiang	2011	1054.93	1050.38	-0.00431

Note: This table compares the NCMS enrollment rates directly reported in the CHSY with those extracted from graphical figures using CorelDRAW. The last column reports the absolute differences between the two sources.

Table A2: Summary Statistics

	Mean	S.D.	Min.	Max.	N
NCMS Variables					
NCMS enrollment rate	0.70	0.35	0.02	1.27	232
Initial NCMS rate in 2004	0.19	0.24	0.02	0.93	232
Inpatient reimbursement rate	0.37	0.10	0.16	0.57	222
Rural Health Resources (per 10,000 people)					
Beds at city hospitals	16	13	5	67	232
Beds at county hospitals	9	4	1	30	232
Beds at CHCs	1	2	0	13	232
Beds at THCs	6	2	0	11	232
Number of city hospitals	0.10	0.07	0.04	0.44	232
Number of county hospitals	0.08	0.05	0.01	0.28	232
Number of CHCs	0.22	0.26	0.02	1.36	232
Number of THCs	0.32	0.16	0.00	0.83	232
Healthcare Utilization (per 10,000 people)					
Ratio of NCMS beneficiaries to enrollment	1.04	1.55	0.02	13.21	232
Total number of outpatient visits	24150	20474	7558	133000	232
Total number of inpatient stays	807	274	310	1771	217

Notes: This Table presents descriptive statistics for key variables used in the analysis from 2004 to 2011. Variables include NCMS enrollment rate, healthcare resources (measured by the number of inpatient beds and institutions per 10,000 people across different facility types), and healthcare utilization indicators. Utilization variables include the number of NCMS claims per rural resident, average outpatient visits, and inpatient service use rate. CHC denotes community health centers, and THC denotes township health centers. County hospitals and THCs mainly serve rural people, while CHCs are mainly used by urban residents.

Table A3: Effects of NCMS Enrollment on Inpatient Care Use After Simultaneously Controlling for The Number of Hospitals and Hospital Beds

	(1)	(2)	(3)	(4)
	Total	CHC	County hospital	THC
NCMS rate	0.041	1.198***	0.079**	-0.006
	(0.055)	(0.417)	(0.032)	(0.137)
THC beds	0.380***	-1.289	0.070	0.991***
	(0.102)	(0.941)	(0.080)	(0.220)
County hospital beds	0.385**	1.203	0.790***	0.198
	(0.164)	(1.343)	(0.140)	(0.416)
CHC beds	-0.011	1.081***	-0.008	0.005
	(0.009)	(0.137)	(0.005)	(0.018)
Number of THCs	-0.239	1.771*	0.019	0.331
	(0.144)	(0.895)	(0.107)	(0.261)
Number of county hospitals	-0.039	-1.014	-0.027	0.125
	(0.114)	(1.150)	(0.092)	(0.295)
Number of CHCs	0.014	0.123	0.018	0.004
	(0.019)	(0.252)	(0.011)	(0.059)
Mean	479.4	0.995	169.1	127.6
R-squared	0.986	0.962	0.995	0.974
Within R-squared	0.411	0.696	0.695	0.681
F-statistic	11.20	110.1	191.4	749.8
Observations	210	210	214	214

Notes: This table reports the effect of NCMS enrollment on inpatient use after simultaneously controlling for both the number of healthcare providers and hospital beds using the baseline model 1. The mean of the dependent variable is the average of inpatient care use in 2004 per 10,000 people and weighted by the rural population in 2003. All estimates are weighted by the rural population in 2003. Standard errors are clustered by province and are shown in parentheses. *** p<0.01, ** p<0.05, * p<0.10.

Table A4: Robustness Check: Effects of NCMS Enrollment on Inpatient Care Use After Controlling for Rural Healthcare Resources: Removing Potential Concurrent Supply Policy

	(1)	(2)	(3)	(4)
	Total	CHC	County hospital	THC
		Panel A. Baseline Estimates		
NCMS rate	0.167**	2.577*	0.110**	0.587**
	(0.079)	(1.295)	(0.046)	(0.221)
Observations	158	158	173	171
Within R-squared	0.311	0.334	0.267	0.443
		Panel B. Number of Beds at Hospitals		
NCMS rate	0.129*	1.447**	0.083*	0.187
	(0.072)	(0.537)	(0.042)	(0.167)
THC beds	0.146	-0.376	0.051	1.028***
	(0.165)	(0.425)	(0.060)	(0.114)
County hospital beds	0.286	0.783	0.709***	0.186
	(0.173)	(0.914)	(0.088)	(0.320)
CHC beds	0.001	1.044***	-0.001	0.008
	(0.011)	(0.126)	(0.007)	(0.019)
Observations	156	156	160	160
Within R-squared	0.397	0.704	0.644	0.674
		Panel C. Number of Healthcare Providers		
NCMS rate	0.171**	2.501**	0.079	0.479**
	(0.065)	(1.040)	(0.055)	(0.178)
Number of THCs	-0.046	1.165	-0.019	1.115***
	(0.059)	(0.972)	(0.050)	(0.157)
Number of county hospitals	0.167	0.463	0.374***	0.125
	(0.124)	(1.001)	(0.096)	(0.217)
Number of CHCs	0.042*	0.764***	0.015	0.074
	(0.024)	(0.259)	(0.018)	(0.052)
Observations	156	156	171	171
Within R-squared	0.375	0.379	0.462	0.594

Notes: Each cell reports estimates of the effects of NCMS enrollment on inpatient care use after controlling for each set of healthcare resources: hospital beds and number of providers in rural areas using the baseline model (1) with full controls of both time-varying demographic covariates and economic covariates for each province, province fixed effects, and region-by-year fixed effects in the period 2004-2009 by removing potential concurrent supply policy after 2009. CHC denotes community health centers, and THC denotes township health centers. Each column corresponds to the estimates of inpatient care at specific hospitals. The mean of the dependent variable is the average of inpatient care use in 2004 per 10,000 people and is weighted by the rural population in 2003. *** p<0.01, ** p<0.05, * p<0.10.

Table A5: Robustness Checks for Supply Responses Using 2004-2008 Data

	(1)<br>County Hospital	(2)<br>CHC	(3)<br>THC
Panel A. Number of Healthcare Providers			
NCMS rate	0.090	-0.112	0.093
	(0.123)	(0.395)	(0.081)
Mean	0.0667	0.105	0.334
Observations	173	173	171
Adjusted R-squared	0.970	0.890	0.974
Within R-squared	0.249	0.164	0.103
F-statistic	6.800	7.075	5.283
Panel B. Number of Beds at Hospitals			
NCMS rate	0.009	1.349	0.339***
	(0.049)	(1.291)	(0.111)
Mean	7.335	0.0976	5.280
Observations	173	162	171
Adjusted R-squared	0.977	0.850	0.881
Within R-squared	0.316	0.403	0.332
F-statistic	31.21	28.43	9.050

Notes: This table provides a robustness check to Table 1. This table report results using data from 2004 to 2008. Each cell reports estimates from the baseline specification (1), with full controls of both time-varying demographic covariates and economic covariates, province fixed effects, and region by year fixed effects on dependent variables per 10,000 people in logarithm form in each panel. CHC denotes community health centers, and THC denotes township health centers. Each column corresponds to the estimates at specific hospitals. The mean of the dependent variable is reported in 2004 per 10,000 people and is weighted by the rural population in 2003. *** p<0.01, ** p<0.05, * p<0.10.

Table A6: Effects of NCMS Enrollment on the Incidence Rate of Infectious Diseases

	(1)	(2)	(3)	(4)
NCMS rate	-144.603**	-136.534*	-122.460*	-85.216
	(66.615)	(69.501)	(63.908)	(69.316)
Inpatient use at THC		0.105		0.088
		(0.191)		(0.146)
Inpatient use at county hospital		-0.073		0.481
		(0.449)		(0.333)
Inpatient use at CHC		-3.106		-5.059*
		(2.546)		(2.872)
Outpatient use at THC			-0.000	-0.003
			(0.013)	(0.012)
Outpatient use at county hospital			-0.041**	-0.062***
			(0.018)	(0.021)
Outpatient use at CHC			0.007	0.014
			(0.009)	(0.010)
Mean	528.1	528.1	528.1	528.1
Observations	210	210	210	210
R-squared	0.924	0.926	0.928	0.933
Within R2	0.275	0.278	0.296	0.328
F-statistic	7.248	6.847	13.06	30.37

Notes: The mortality and disease incidence is from the Chinese CDC, which monitors and records the incidence of various infectious diseases, including respiratory infections like influenza and pneumonia, vector-borne diseases such as malaria and dengue fever, gastrointestinal diseases like hepatitis and foodborne infections, sexually transmitted infections including HIV, vaccine-preventable diseases like measles, and emerging diseases like COVID-19 and avian influenza. Since the CDC does not report these figures by healthcare provider, we present the evidence by controlling for both inpatient care (column 2), outpatient care (column 3), and both types of care combined (column 4). *** p<0.01, ** p<0.05, * p<0.10.

Table A7: Robustness Check: Heterogeneous Supply Responses Across Regions with Differential Healthcare Supply

	(1)	(2)	(3)	(4)	(5)	(6)	(7)	(8)	(9)
		County Hospital			CHC			THC	
	West	Middle	East	West	Middle	East	West	Middle	East
					Panel A: Number of Healthcare Providers				
NCMS rate	0.061	0.412**	0.308**	0.164	0.095	-0.035	0.047	-0.149*	-0.002
	(0.231)	(0.161)	(0.116)	(0.613)	(1.011)	(0.426)	(0.043)	(0.068)	(0.074)
Observations	88	64	79	88	64	79	88	64	73
Adjusted R-squared	0.964	0.989	0.969	0.895	0.919	0.809	0.992	0.982	0.919
Within R-squared	0.295	0.766	0.532	0.243	0.563	0.193	0.185	0.677	0.249
Mean	0.0667	0.0667	0.0667	0.105	0.105	0.105	0.334	0.334	0.334
					Panel B: Number of Beds at Hospitals				
NCMS rate	0.135	0.102	0.034	1.444	-4.249	2.188*	0.360**	-0.340***	0.114
	(0.186)	(0.110)	(0.054)	(1.531)	(2.582)	(1.095)	(0.131)	(0.084)	(0.097)
Observations	88	64	79	79	64	77	88	64	73
Adjusted R-squared	0.971	0.975	0.992	0.933	0.913	0.841	0.972	0.960	0.795
Within R-squared	0.226	0.464	0.674	0.427	0.552	0.488	0.608	0.385	0.436
Mean	7.335	7.335	7.335	0.0976	0.0976	0.0976	5.280	5.280	5.280

Notes: Each cell reports estimates from the baseline specification (1), with full controls of both time-varying demographic covariates and economic covariates, province fixed effects, and region by year fixed effects on dependent variables per 10,000 people in logarithm form in each panel. CHC denotes community health centers, and THC denotes township health centers. County hospitals and THCs mainly serve rural people, while CHCs are mainly used by urban residents. West region includes provinces: Chongqing, Gansu, Guangxi, Guizhou, Inner Mongolia, Ningxia, Qinghai, Shaanxi, Sichuan, Xinjiang, and Yunnan. Middle region inlcudes provinces: Anhui, Henan, Hubei, Hunan, Heilongjiang, Jilin, Jiangxi, and Shanxi. East region include provinces: Beijing, Fujian, Guangdong, Hainan, Hebei, Jiangsu, Liaoning, Shandong, Shanghai, and Zhejiang. The mean of each dependent variable is the average in 2004 per 10,000 people and is weighted by the rural population in 2003. All estimates are weighted by the rural population in 2003. Standard errors are clustered by province and are shown in parentheses. *** $p<0.01$, ** $p<0.05$, * $p<0.10$.

Table A8: Robustness Check: Heterogeneous Supply Responses Across Rural and Urban Provinces

	(1)	(2)	(3)	(4)	(5)	(6)
	County Hospital		CHC		THC	
	Rural	Urban	Rural	Urban	Rural	Urban
	Panel A: Number of Healthcare Providers					
NCMS rate	0.323	0.112*	0.596***	-0.692*	0.067	0.069
	(0.195)	(0.054)	(0.159)	(0.347)	(0.078)	(0.114)
Observations	112	119	112	119	112	113
Adjusted R-squared	0.927	0.987	0.942	0.642	0.989	0.932
Within R-squared	0.228	0.517	0.364	0.161	0.683	-0.0866
Mean	0.0667	0.0667	0.105	0.105	0.334	0.334
	Panel B: Number of Beds at Hospitals					
NCMS rate	0.097	0.074	3.637***	-0.544	0.534***	0.152
	(0.111)	(0.067)	(0.816)	(0.912)	(0.114)	(0.171)
Observations	112	119	105	115	112	113
Adjusted R-squared	0.961	0.984	0.935	0.823	0.965	0.785
Within R-squared	0.118	0.436	0.520	0.513	0.734	0.215
Mean	7.335	7.335	0.0976	0.0976	5.280	5.280

Notes: Each cell reports estimates from the baseline specification (1), with full controls of both time-varying demographic covariates and economic covariates, province fixed effects, and region by year fixed effects on dependent variables per 10,000 people in logarithm form in each panel. CHC denotes community health centers, and THC denotes township health centers. County hospitals and THCs mainly serve rural people, while CHCs are mainly used by urban residents. Rural province is defined that the the share of rural population is above the median in year 2003. The mean of each dependent variable is the average in 2004 per 10,000 people and is weighted by the rural population in 2003. All estimates are weighted by the rural population in 2003. Standard errors are clustered by province and are shown in parentheses. *** p<0.01, ** p<0.05, * p<0.10.

Table A9: Robustness Checks for Supply Responses Using Alternative Transformations of the Dependent Variable

	Log+constant			IHS Transformation		
	(1)	(2)	(3)	(4)	(5)	(6)
	County Hospital	CHC	THC	County Hospital	CHC	THC
	Panel A. Number of Healthcare Providers					
NCMS rate	0.004	-0.114*	0.006	0.004	-0.160*	0.008
	(0.009)	(0.060)	(0.017)	(0.010)	(0.079)	(0.022)
Mean	0.0667	0.105	0.334	0.0667	0.105	0.334
Observations	231	231	231	231	231	231
Adjusted R-squared	0.968	0.847	0.981	0.968	0.839	0.980
Within R-squared	0.355	0.181	0.301	0.356	0.194	0.290
F-statistic	8.795	7.841	1.274	8.456	7.783	1.204
	Panel B. Number of Beds at Hospitals Providers					
NCMS rate	0.021	-0.031	0.204*	0.028	-0.068	0.225*
	(0.037)	(0.146)	(0.105)	(0.042)	(0.195)	(0.127)
Mean	7.335	0.0976	5.280	7.335	0.0976	5.280
Observations	231	231	231	231	231	231
Adjusted R-squared	0.976	0.905	0.898	0.976	0.900	0.890
Within R-squared	0.306	0.297	0.454	0.297	0.305	0.438
F-statistic	16.94	9.634	14	14.13	8.962	10.22

Notes: This table provides a robustness check to Table 1. Each cell reports estimates from the baseline specification (1), with full controls of both time-varying demographic covariates and economic covariates, province fixed effects, and region by year fixed effects on dependent variables per 10,000 people in logarithm form in each panel. The dependent variables—measured per 10,000 people—are transformed using (i) log(y + 0.01) and (ii) the inverse hyperbolic sine transformation, as alternatives to the logarithmic specification used in the main analysis. CHC denotes community health centers, and THC denotes township health centers. Each column corresponds to the estimates at specific hospitals. The mean of the dependent variable is reported in 2004 per 10,000 people and is weighted by the rural population in 2003. *** p<0.01, ** p<0.05, * p<0.10.

Table A10: Effect of NCMS Enrollment on Healthcare Investments by Rural Medical Providers: Alternative Specification Without Controls

	(1) County hospital	(2) CHC	(3) THC
	Panel A. Number of Healthcare Providers		
NCMS rate	0.301**	-0.863	0.452
	(0.123)	(0.564)	(0.329)
Mean	0.067	0.105	0.334
Observations	232	232	226
Adjusted R-squared	0.975	0.817	0.898
Within R-squared	0.088	0.068	0.083
F-statistic	6.038	2.340	1.887
	Panel B. Number of Beds at Hospitals		
NCMS rate	0.209**	1.346	0.800**
	(0.080)	(0.982)	(0.385)
Mean	7.335	0.098	5.280
Observations	232	221	226
Adjusted R-squared	0.982	0.816	0.685
Within R-squared	0.095	0.041	0.171
F-statistic	6.897	1.877	4.317

Notes: This table provides a robustness check to baseline Table 1. Each cell reports estimates from the baseline specification (1), without any controls of time-varying demographic covariates and economic covariates, with province fixed effects, and region by year fixed effects on dependent variables per 10,000 people in logarithm form in each panel. CHC denotes community health centers, and THC denotes township health centers. County hospitals and THCs mainly serve rural people, while CHCs are mainly used by urban residents. The mean of each dependent variable is the average in 2004 per 10,000 people and is weighted by the rural population in 2003. All estimates are weighted by the rural population in 2003. Standard errors are clustered by province and are shown in parentheses. *** p<0.01, ** p<0.05, * p<0.10.

Table A11: Effects of NCMS Enrollment on Inpatient Care Use After Controlling for Rural Healthcare Resources: Alternative Specification Without Controls

	(1) Total	(2) CHC	(3) County hospital	(4) THC
	Panel A. Baseline Estimates			
NCMS rate	0.192***	2.522***	0.242***	1.103**
	(0.047)	(0.600)	(0.060)	(0.527)
Mean	479.4	0.99	169.1	127.6
Adjusted R-squared	0.969	0.837	0.986	0.831
Within R-squared	0.118	0.125	0.128	0.170
Observations	217	217	232	226
	Panel B. Number of Beds at Hospitals			
NCMS rate	0.097**	1.653***	0.051	0.053
	(0.041)	(0.492)	(0.053)	(0.162)
THC beds	0.053	-0.287*	0.035	1.206***
	(0.044)	(0.155)	(0.021)	(0.042)
County hospital beds	0.387**	0.714	0.747***	0.262
	(0.153)	(0.775)	(0.075)	(0.240)
CHC beds	-0.003	1.002***	-0.010	0.009
	(0.011)	(0.118)	(0.007)	(0.021)
Observations	211	211	215	215
Adjusted R-squared	0.973	0.928	0.994	0.960
Within R-squared	0.297	0.622	0.622	0.810
	Panel C. Number of Healthcare Providers			
NCMS rate	0.151***	2.311***	0.103*	0.411**
	(0.047)	(0.697)	(0.051)	(0.200)
Number of THCs	0.022	0.026	0.028	1.354***
	(0.022)	(0.346)	(0.019)	(0.052)
Number of county hospitals	0.171	0.256	0.342***	0.331
	(0.114)	(0.720)	(0.082)	(0.243)
Number of CHCs	0.015	0.139	-0.010	-0.018
	(0.015)	(0.270)	(0.017)	(0.065)
Observations	211	211	226	226
Adjusted R-squared	0.967	0.825	0.989	0.944
Within R-squared	0.149	0.080	0.287	0.726

Notes: This table provides a robustness check to Table 2. Each cell reports estimates of the effects of NCMS enrollment on inpatient care use after controlling for each set of healthcare resources: hospital beds and number of providers in rural areas using the baseline model (1) without any controls of time-varying demographic covariates and economic covariates for each province, with province fixed effects, and region-by-year fixed effects. CHC denotes community health centers, and THC denotes township health centers. Each column corresponds to the estimates of inpatient care at specific hospitals. The mean of the dependent variable is the average of inpatient care use in 2004 per 10,000 people and is weighted by the rural population in 2003. *** p<0.01, ** p<0.05, * p<0.10.

Table A12: Robustness Checks for Supply Responses Using Alternative Specifications

	Only FE			FE + covariates			FE + covariates + weights		
	(1)	(2)	(3)	(4)	(5)	(6)	(7)	(8)	(9)
	County Hospital	CHC	THC	County Hospital	CHC	THC	County Hospital	CHC	THC
Panel A. Number of Healthcare Providers									
NCMS rate	0.301**	-0.863	0.452	0.168	-0.038	0.267	0.130	-0.108	0.062
	(0.123)	(0.564)	(0.329)	(0.099)	(0.337)	(0.206)	(0.095)	(0.280)	(0.074)
Mean	0.0667	0.105	0.334	0.0667	0.105	0.334	0.0667	0.105	0.334
Observations	232	232	226	231	231	225	231	231	225
Adjusted R-squared	0.975	0.817	0.898	0.979	0.853	0.922	0.963	0.879	0.970
Within R-squared	0.0877	0.0678	0.0828	0.242	0.246	0.209	0.286	0.120	0.197
F-statistic	6.038	2.340	1.887	4.473	14.71	2.532	5.608	8.027	6.060
Panel B. Number of Beds at Hospitals									
NCMS rate	0.209**	1.346	0.800**	0.054	0.735	0.497*	0.029	1.046	0.282**
	(0.080)	(0.982)	(0.385)	(0.045)	(0.839)	(0.249)	(0.043)	(0.941)	(0.109)
Mean	7.335	0.0976	5.280	7.335	0.0976	5.280	7.335	0.0976	5.280
Observations	232	221	226	231	220	225	231	220	225
Adjusted R-squared	0.982	0.816	0.685	0.987	0.844	0.761	0.977	0.862	0.892
Within R-squared	0.0947	0.0407	0.171	0.354	0.192	0.274	0.294	0.373	0.379
F-statistic	6.897	1.877	4.317	10.13	3.297	6.158	13.62	15.62	11.46

Notes: This table provides a robustness check to Table 1. The dependent variables are number of medical institutions and hospitals beds per 10,000 people in Panel A and Panel B, respectively. Columns 1-3 report estimates using by only including province fixed effects, and region by year fixed effects; columns 4-6 additionally controls for time-varying demographic covariates and economic covariates; columns 7-9 additionally weight the regression by rural population in 2003. CHC denotes community health centers, and THC denotes township health centers. Each column corresponds to the estimates at specific hospitals. The mean of the dependent variable is reported in 2004 per 10,000 people and is weighted by the rural population in 2003. *** p<0.01, ** p<0.05, * p<0.10.
